# POCUS Allows for Rapid Elucidation of Acute Kidney Injury in a Patient with Progressive Multiple Myeloma

**DOI:** 10.24908/pocus.v7iKidney.15342

**Published:** 2022-02-01

**Authors:** Liann Abu Salman, Nathaniel Reisinger

**Affiliations:** 1 Division of Renal, Electrolytes and Hypertension, University of Pennsylvania Philadelphia, PA USA

**Keywords:** multiple myeloma, plasmacytoma, acute kidney injury, hydronephrosis, POCUS

## Abstract

A 63-year-old man with past history of multiple myeloma recently started on a regimen of daratumumab, carfilzomib, and dexamethasone was referred to our emergency department for a rapidly rising serum creatinine as high as 10 mg/dL. He complained of fatigue, nausea, and poor appetite. Exam revealed hypertension, but no edema or rales. Labs were consistent with AKI without hypercalcemia or evidence of hemolysis or tumor lysis. Urinalysis and urine sediment were bland without proteinuria, hematuria, or pyuria. Initial concern was for hypovolemia or myeloma cast nephropathy. POCUS revealed no overt evidence of volume overload or depletion, instead revealing bilateral hydronephrosis. Bilateral percutaneous nephrostomies were placed with resolution of the AKI. Ultimately, referral imaging revealed interval progression of bulky retroperitoneal extramedullary plasmacytomas compressing the ureters bilaterally related to the underlying multiple myeloma.

## Case

A 63-year-old man with past medical history of advanced IgG Kappa multiple myeloma presented with worsening fatigue, nausea, and loss of appetite. He had progression of disease despite completion of multiple prior therapies for myeloma including autologous stem cell transplant and three weeks prior to presentation was started on combination therapy with daratumumab, carfilzomib, and dexamethasone. Subsequently, he had waxing and waning levels of energy and decreased oral intake. He denied lightheadedness, abdominal pain, vomiting, rash, and joint pain. He denied any urinary complaints and noted no change in urine output. He noted some loose stools. He had weekly outpatient labs and was noted to have a rising serum creatinine (Cr) from 1.6 mg/dL to 3.8 mg/dl one week prior to presentation. Repeat labs the day of presentation demonstrated a Cr of 9.3mg/dL and the patient was instructed to present to the emergency department for further evaluation. 

On arrival, the patient’s vitals were: blood pressure 176/109 mmHg, heart rate 64 beats per minute, oxygen saturation 99% on room air, and temperature of 36.9C. Physical exam demonstrated moist mucous membranes and good skin turgor. Jugular veins were not well-visualized. Axillary sweat was present. Pulmonary crackles and lower extremity edema were absent. There was no costovertebral angle tenderness. 

Initial labs confirmed elevated blood urea nitrogen (91mg/dl) and creatinine levels (10.76 mg/dl). Other labs were notable for serum sodium of 138 mmol/dL, potassium elevated at 5.5 mmol/dL, bicarbonate low at 17 mmol/dL, calcium normal at 8.7 mg/dL (with normal albumin), and phosphate elevated at 8.7 mg/dL. Lactate dehydrogenase and uric acid were only mildly elevated at 200 units per liter and 10.8 mg/dL respectively. Complete blood count revealed white blood cells of 4400 per µL, hemoglobin of 10.3 g/dL, and platelets of 6300 per µL. Urinalysis was negative for heme, albumin, and leukocyte esterase with bland sediment. Quantitative serum kappa free light chains were 203.8 mg/L and quantitative serum lambda free light chains were 6.5 mg/L with kappa to lambda ratio of 31.4 roughly unchanged from the week prior. Repeat immunoglobulin levels and serum protein electrophoresis revealed immune paresis with a stable M spike in the gamma region consistent with known IgG kappa band and stable from prior. 

**Figure 1:**
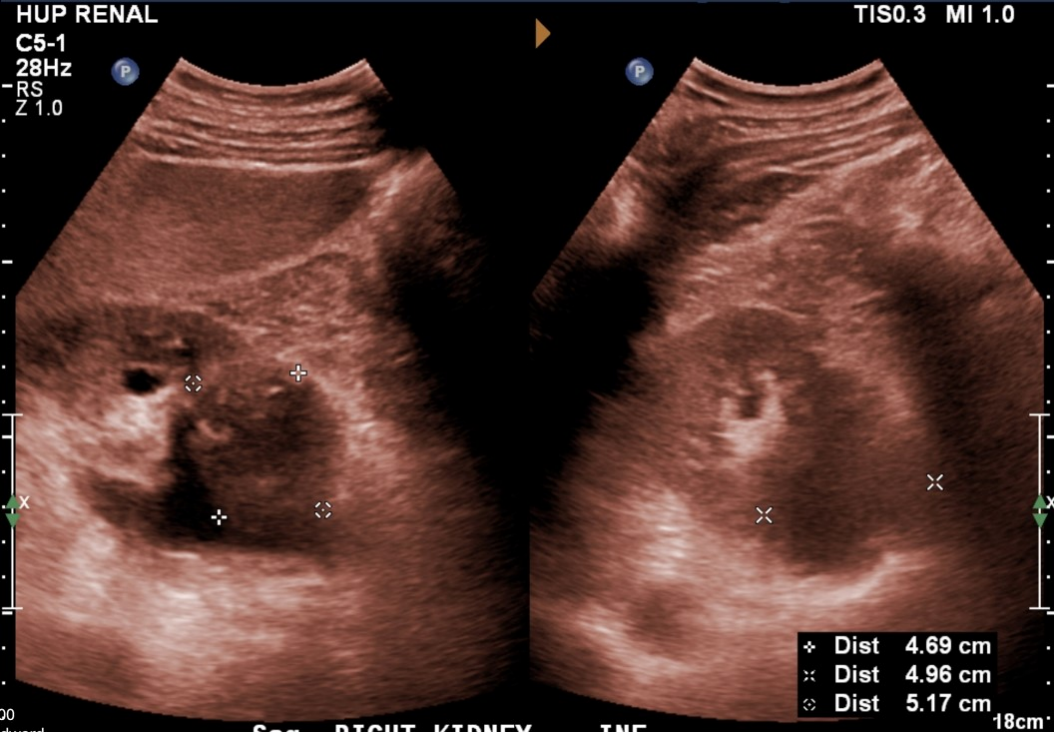
Confirmatory referral kidney ultrasound by radiology in sepia tone demonstrating hypoechoic 5.2 cm retroperitoneal lesions likely representing extramedullary plasmacytomas compressing the proximal ureter. Left: right kidney long axis. Right: right kidney short axis.

**Figure 2:**
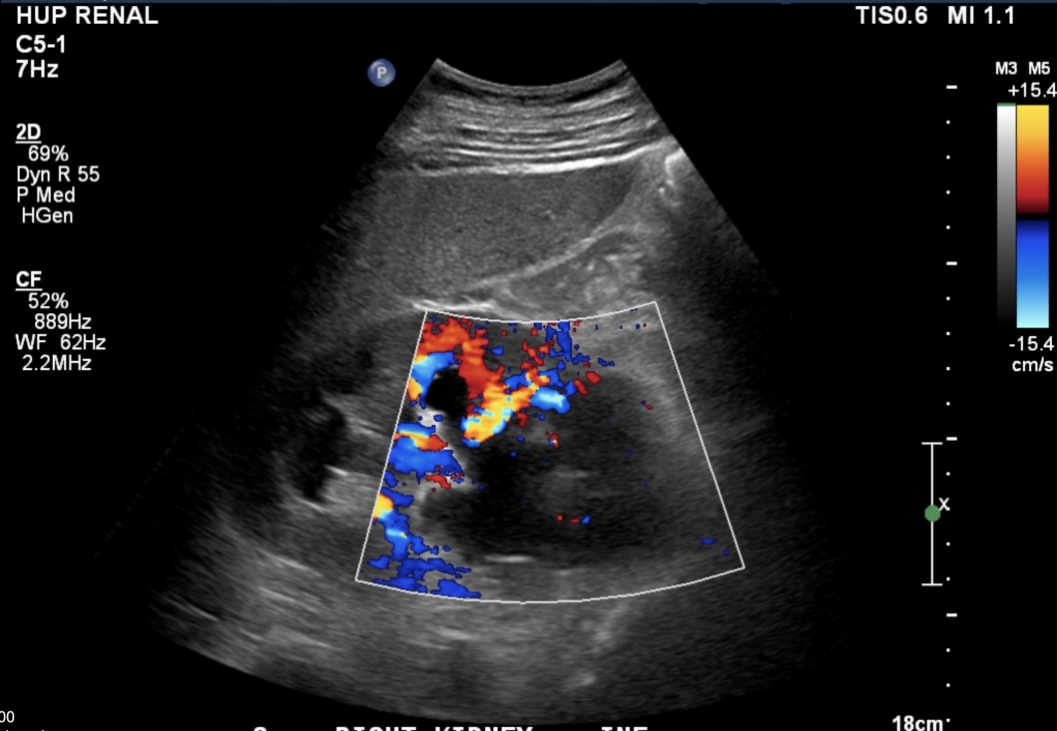
Doppler imaging of right kidney, long axis demonstrating absence of flow in the dilated renal pelvis, proximal ureter, and masses in the retroperitoneum at the inferior pole.

**Figure 3:**
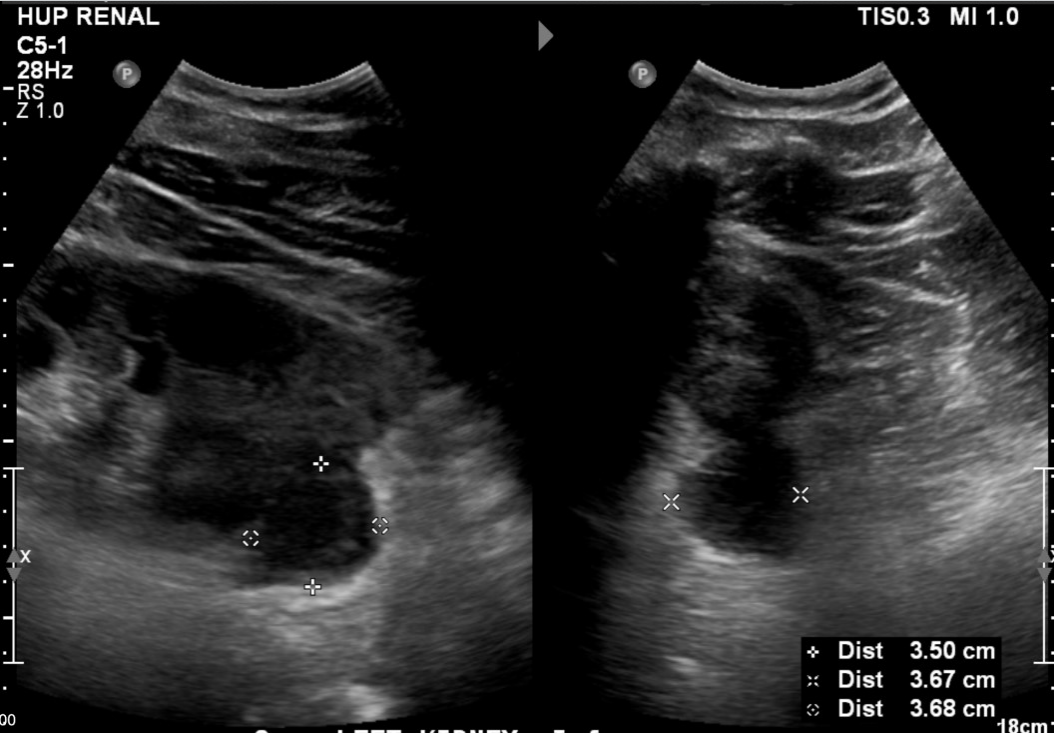
Figure 3. Referral kidney ultrasound of left kidney in long axis demonstrating suspected plasmacytomas 3.7 cm in maximum diameter at the inferior pole. Left: left kidney long axis. Right: left kidney short axis.

**Figure 4:**
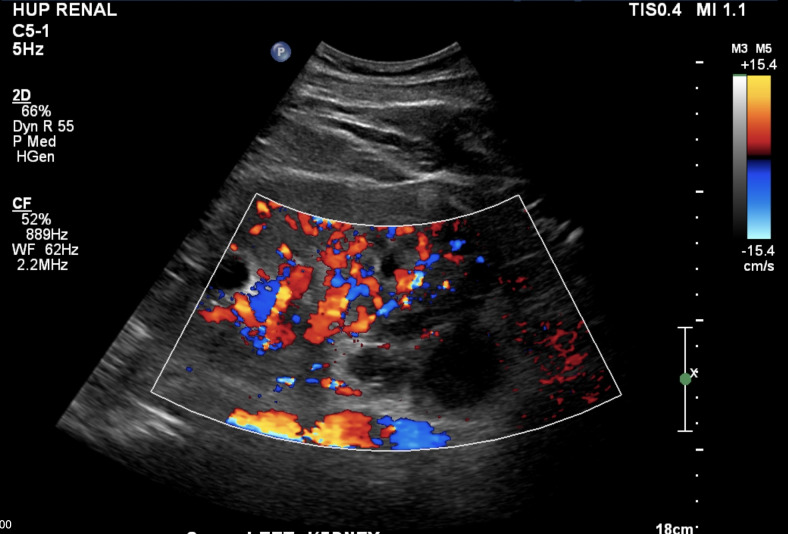
Doppler imaging of left kidney in long axis demonstrating lack of fast flow in the area of suspected plasmacytomas.

Point of care ultrasound including focused cardiac, lung, and bilateral kidney ultrasound was undertaken. Lung included diffuse A-line pattern in all visualized fields and no pleural effusions (Video S1). Focused cardiac assessment revealed a normal-sized inferior vena cava that collapsed less than 50% with inspiration (Video S2).The right internal jugular vein was undistended 2 centimeters above the sternal angle with the patient in the supine position. (Video S3). Kidney ultrasound revealed enlarged kidneys bilaterally with bilateral hydronephrosis. (Video S4-6 and Figure 1-4). 

On ultrasound, hydronephrosis appears as confluent anechoic areas representing urine in a dilated renal pelvis and calyces 1. In mild hydronephrosis, the renal pelvis and calyces are dilated but the cortex and pelvicalyceal pattern is unaffected. As the hydronephrosis increases in severity, the medullary pyramids flatten and the pelvicalyceal system dilates leading to outpouching of the calyces. In severe hydronephrosis, the renal pelvis may appear ballooned and the cortico-medullary differentiation is lost leading to a thin cortex 2.

**Figure 5:**
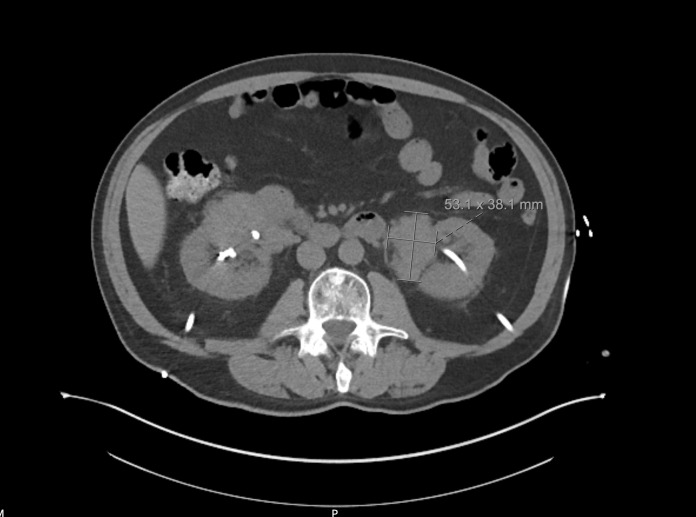
Computed tomography, coronal view in the abdomen demonstrating interval placement of bilateral percutaneous nephrostomies. On the left the plasmacytomas measure 5.3 cm in maximum diameter.

**Figure 6:**
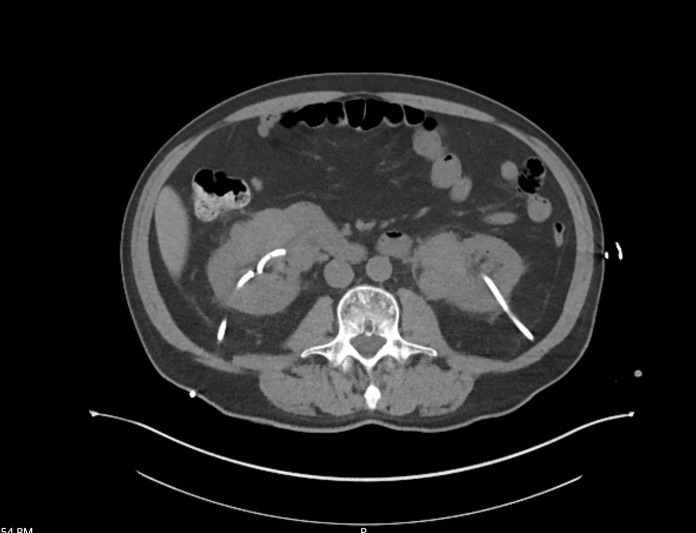
Computed tomography, coronal view in the abdomen demonstrating bilateral kidneys with nephrostomies in place and bulky plasmacytomas at inferomedial border of kidneys bilaterally.

On review of the patient’s prior imaging, he was noted to have retroperitoneal extramedullary plasmacytomas on a computed tomography scan done six weeks prior. He had interval increase of the plasmacytomas despite initiation of chemotherapy leading to obstruction. Despite the temporal association between our patient starting chemotherapy and the development of acute kidney injury, it was not in fact a causal relationship. The use of POCUS revealed bilateral hydronephrosis and guided our management. We recommended bilateral percutaneous nephrostomy (PCN) insertion as both a diagnostic and therapeutic maneuver. Within 12 hours of PCN placement, his Cr started to decline and within 72 hours his renal function returned to his prior baseline. Ultimately repeat computed tomography demonstrated interval increase in size of retroperitoneal extramedullary plasmacytomas (Figure 5, 6).

When evaluating acute kidney injury (AKI) in multiple myeloma patients the differential diagnosis is wide. Most commonly, myeloma cast nephropathy is suspected. Other causes include deposition diseases, proximal tubulopathy, or direct nephrotoxicity from the various chemotherapeutic and immunotherapy agents used 3. Acute kidney injury may also arise from side effects of the chemotherapeutic agents which often include nausea, vomiting, and decreased oral intake leading to pre-renal insults or even acute tubular necrosis. Given the bland nature of the urine sediment and lack of physical exam or ultrasonographic evidence of extremes of volume overload our initial differential diagnosis included myeloma cast nephropathy, obstructive nephropathy, and thrombotic microangiopathy due to carfilzomib therapy. 

Extramedullary plasmacytomas rarely occur during the course of multiple myeloma with an incidence rate of around 4% 4. When present, they are most frequently seen along the respiratory tract, lymph nodes and skin and soft tissue. Involvement of the urinary tract is quite uncommon with seven cases involving the renal parenchyma and only two cases of urinary tract obstruction reported in the literature 5, 6. When evaluating acute kidney injury, a wide differential should be sought, and obstructive nephropathy should be considered. As POCUS is inexpensive and readily available, it should be used in the evaluation of AKI as prompt intervention can lead to marked improvement and even resolution of kidney injury.

## Patient Consent

The authors certify that informed consent was obtained for the clinical information and images reported.

## Disclosures

None.

## Supplementary Material

 Video S1 Representative point-of-care lung ultrasound video with intact lung sliding and A-line pattern with no evidence of pulmonary congestion.

Video S2 Infradiaphragmatic inferior vena cava in transverse view. Note that the IVC is normal in size and collapses less that 50% with inspiration. Hepatic veins can be seen draining into the IVC.

Video S3 Right internal jugular vein in transverse view with right common carotid artery posteriorly. Note that the IJ is collapsed approximately 2 cm above the level of the sternal angle.

Video S4 Right kidney long axis view. Note the dilated renal pelvis with confluent anechoic spaces within the hyperechoic medullary sinus fat. Major calyces are dilated. Hypoechoic masses are just visible encasing the proximal ureter at the inferior pole. The liver is hyperechoic likely representing hepatic infiltration by paraproteins.

Video S5 Right kidney short axis view confirming the presence of hydronephrosis and recapitulating hypoechoic structures at the inferior pole.

Video S6 Left Kidney long axis view. Again note the anechoic spaces confluent with renal pelvis within the medullary sinus fat representing hydronephrosis.
